# Probing Protein Secondary Structure Influence on Active Centers with Hetero Two-Dimensional Correlation (Resonance) Raman Spectroscopy: A Demonstration on Cytochrome C

**DOI:** 10.1177/00037028211028916

**Published:** 2021-07-09

**Authors:** Julian Hniopek, Thomas Bocklitz, Michael Schmitt, Jürgen Popp

**Affiliations:** 1 Department of Spectroscopy/Imaging, Leibniz-Institute of Photonic Technologies, Jena, Germany; 2 Institute of Physical Chemistry & Abbe Center of Photonics, 9378 Friedrich Schiller University Jena, Jena, Germany; 3 Department of Photonic Data Science, Leibniz-Institute of Photonic Technologies, Jena, Germany

**Keywords:** Two-dimensional correlation spectroscopy, 2D-COS, structure–function relationship, resonance Raman spectroscopy, Raman spectroscopy

## Abstract

The functionality of active centers in proteins is governed by the secondary and higher structure of proteins which often lead to structures in the active center that are different from the structures found in protein-free models of the active center. To elucidate this structure–function relationship, it is therefore necessary to investigate both the protein structure and the local structure of the active center. In this work, we investigate the application of hetero (resonance) Raman two-dimensional correlation spectroscopy (2D-COS) to this problem. By employing a combination of near-infrared-Fourier transform-Raman- and vis-resonance Raman spectroscopy, we could show that this combination of techniques is able to directly probe the structure–function relationship of proteins. We were able to correlate the transition of the heme center in cytochrome c from low to high spin with changes in the secondary structure with the above mentioned two spectroscopic in situ techniques and without sample preparation. Thereby, we were able to reveal that the combination of a spectroscopic method to selectively observe the active center with a technique that monitors the whole system offers a promising toolkit to investigate the structure–function relationship of proteins with photoactive centers in general.

## Graphical abstract

**Graphical Abstract:**
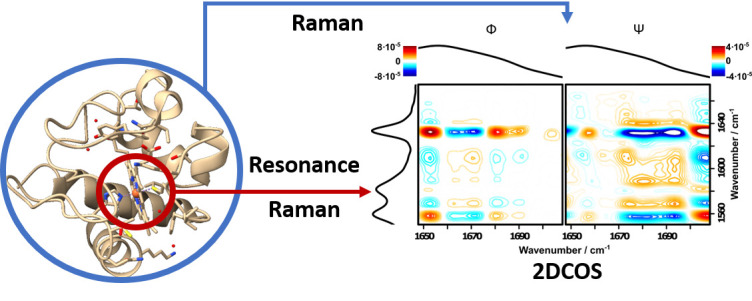

## Introduction

Proteins, together with nucleic acids, lipids, and glycans, are the building blocks that make life possible.^1^ They serve as structural building blocks, transport systems, cell signaling compounds in all living organisms and, as enzymes, enable reactions that are necessary for life. Their functionality is highly governed by their specific structure, a concept known as the structure–function relationship. Specifically for proteins, this does not only encompass their molecular composition or the order of chemical bonds (i.e., structural isomery), but is also highly dependent on supermolecular structural elements known as secondary, tertiary, and quarternary structure.^2^ The specific arrangement of proteins into these structures is essential for the wide range of functions they can fulfill.^3,4^

Many proteins, which do not merely serve as structural elements, contain an active center that often includes metal ions crucial to enable the protein’s role in its biological system. Well-known proteins with such an active site are for instance chlorophyll (which contains an Mg^2+^-porphyrin center),^5^ photosystem II (involving a manganese cluster structure),^6^ or the heme group proteins, which have an active core containing iron-porphyrine system.^7^ While some of these active centers can be synthesized in vitro, they often show none or at least drastically reduced activity compared to their native protein environment, demonstrating the importance of the protein structure to its function.^3,4^ To elucidate the function of a reactive protein, it is therefore imperative to investigate its supermolecular structure as well as the active center to find structure–function relationships. However, investigating the active center of a protein is often comparatively difficult, since it is usually small compared to the rest of the protein and is often well shielded from the outside by amino acid structures. This complicates targeted investigation of the active site with unspecific techniques such as nuclear magnetic resonance (NMR) spectroscopy.

Raman spectroscopy is an excellent tool to investigate the supermolecular structure of proteins.^8^ The Raman spectrum and here especially the vibrations of the amide groups in the peptide bonds of the protein is highly dependent on the secondary structure of the protein.^8,9^ This enables the detection of supermolecular building blocks of the protein. Furthermore, Raman spectroscopy is a noninvasive method, does in general not require complex sample preparation and is insensitive to water, which enables the observation of proteins in situ.^10^ Since protein structures usually show a large dependency on environmental conditions, this is especially important to resolve the structure–function relationships of proteins in their native environment.

As discussed above, protein active centers often contain metal centers, which are photoactive in the visible or near-ultraviolet spectral region, making them accessible to resonance Raman spectroscopy. In contrast to the non-resonance Raman effect, where the inelastic scattering process takes place via a virtual state, the resonance Raman effect involves a real electronic state.^11^ This coupling of the scattering process to a real electronic transition leads to a multifold enhancement of the scattering efficiency for vibrational modes associated with the electronic transition.^12^ This property leads to two distinct advantages for resonance Raman spectroscopy concerning the study of active protein centers: First, the signal enhancement oftentimes is necessary to be able to observe the active center, since its “concentration” inside the protein environment is very low. Second, the detected scattering processes are selective to the active center, since the vibrational modes have to be coupled to the electronic transition in the active center to experience enhancement.^13^

Combining both non- and resonance Raman spectroscopy thereby enables a simultaneous observation of the secondary structure of the protein as well as the local structure of the active center. In doing so, two-dimensional correlation spectroscopy (2D-COS) offers an ideal tool to analyze the results of multiple spectroscopic techniques and to investigate the correlation between observations in both spectra.^14–17^ 2D-COS recovers the cross-correlation function in data sets recorded under external perturbation and therefore enables the separation of correlated changes from uncorrelated ones. It is possible to extend 2D-COS to use two spectroscopic techniques (hetero 2D-COS),^15^ which then gives a direct way to investigate the relationship of spectral changes between different techniques. This approach has especially been used to combine near- with mid-infrared spectroscopy, where the mid-infrared spectra offer a way to help assign changes in the near-infrared spectrum, which in turn gives further insight into the structure of the samples by analyzing the various combination bands.^18,19^ Applying this approach to Raman/resonance Raman should make it possible to elucidate correlated changes of the active center and protein structure under an external perturbation and identify structure–function relationships.

**Figure 1.**
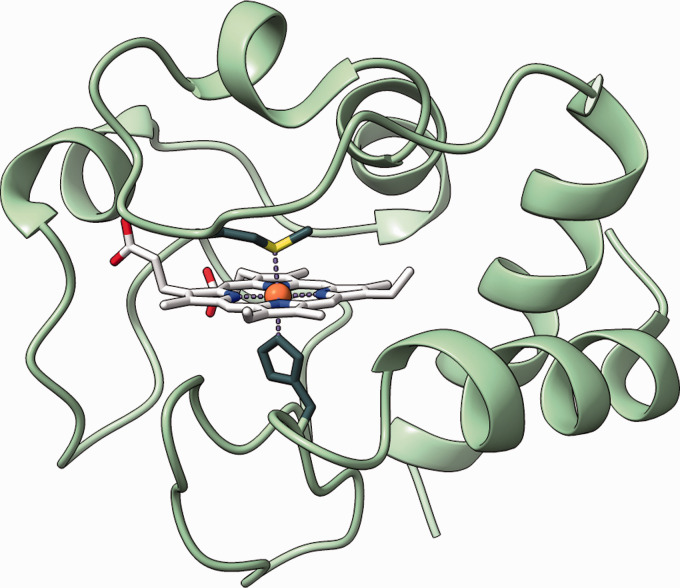
Crystal structure of bovine heart cytochrome c in its native state. Crystal structure obtained from PDB—Number: 2B4Z and reproduced using UCSF ChimeraX.^20–22^ The porphyrin moiety is marked in light gray, while the stabilizing His and Met ligands are displayed in dark gray. The protein backbone is colored light green.

We explored the application of this hetero 2D-COS approach to cytochrome c, a heme-type protein that plays a central role in the energy metabolism of all living cells, specifically in the electron transport chain inside mitochondria. Cytochrome c contains a heme c moeity, an Fe^III^-porphyrin complex (light gray structure in Fig. 1), as its active center. This complex can undergo reversible changes in redox state (reduction to Fe^II)^ and thereby store and transfer electrons in the electron transport chain.

Conversely to free heme c, in which the Fe^III^ is in a high-spin form (the five d electrons are unpaired and occupy t 2g and e g molecular orbitals), the iron center in cytochrome c is stabilized in its low-spin form (two pairs of d electrons and one unpaired d electron, all in t 2g orbitals) by coordination of one His and one Met ligand (dark gray structures in Fig. 1).^21^ This low-spin configuration is imperative for the function of cytochrome c since it minimizes the Marcus reorganization energy of the active center during the redox processes.^23^

The presence of these ligands is therefore necessary for the function of cytochrome c, a prominent example of a protein structure–function relationship. Changes of the secondary structure, e.g., induced by denaturation of the protein with CaCl_2_ will therefore drastically influence the functionality of this protein.^24,25^ Cytochrome c has been studied extensively using resonance Raman spectroscopy in the past and is a very well characterized system,^26–29^ which makes it an ideal system to demonstrate the applicability and potential of the herewith reported (resonance) Raman hetero 2D-COS approach to elucidate structure–function relationships in photo-active proteins.

## Methods

### Raman Spectroscopic Measurements

Solutions of cytochrome c (from bovine heart, Sigma Aldrich, Germany) in water containing various amounts of calcium chloride as denaturating agent were prepared by dissolving 8 mg cytochrome c in 100 µL of water or in a calcium chloride solution with concentrations of 0.5 to 5 M in steps of 0.5 M. The solutions were transferred into a quartz cuvette and sealed tightly with a polytetrafluoroethylene (PTFE) stopper and parafilm.

Non-resonance Raman-spectroscopic measurements were performed on a multispec Fourier transform- (FT) Raman spectrometer (Bruker Corporation, Massachusetts) in the range between 100 and 4000 cm^−1^ with a spectral resolution of 4 cm^−1^. The Raman excitation light at 1064 nm was provided using a neodymium-doped yttrium aluminum garnet (Nd:YAG) laser (Klastech DeniCAFC-LC-3/40, Germany). The laser power was set to 1000 mW. The laser light was focused into the quartz cuvette containing the solution of cytochrome c and various amounts of calcium chloride. The Raman scattered light was detected in a 180° scattering geometry using liquid nitrogen cooled Ge-detector. To improve the signal-to-noise ratio, 2048 scans were averaged to record the final Raman spectrum.

The resonance Raman spectra were collected on a LabRAM HR inv (Horiba Jobin Yvon, Germany) confocal Raman microscope in the range between 600 and 1800 cm^−1^. The laser light at 532 nm provided by a frequency doubled diode pumped Nd:YAG laser was focused into a quartz cuvette containing the sample solution via a 10 × Objective (NA: 0.25, MPlan 10 × , Olympus Corporation, Japan). The laser power at the sample plane was attenuated with a gray filter to 8 mW. The scattered light was passed through two notch filters (cutoff ± 200 cm^−1^) to separate the Raman- from the Rayleigh-scattered light and dispersed with a 1800 g/mm reflection grating (spectral resolution: ∼2 cm^−1^) before being detected by a thermo-electrically cooled charge-coupled device (CCD) (T Op = 220 K, Synapse 1024 × 256, Horiba Jobin Yvon, Germany). The resonance Raman spectra were collected over 30 s and 40 accumulations for each spectrum were averaged to record the final resonance Raman spectrum.

### Data Processing and Two-Dimensional Correlation Spectroscopy

All recorded Raman spectra were analyzed using GNU R (v.4.0.2).^30^ The raw Raman spectra were restricted to the fingerprint regions (600 to 1800 cm^−1^) and background corrected using the sensitive nonlinear iterative peak (SNIP) algorithm (iterations: 60).^31^ The FT-Raman spectra were normalized to the area of the amide-I region (1620 to 1700 cm^−1^) to account for the varying scattering cross-sections of cytochrome c in the various states of degradation. This normalization enables a comparison of the relative changes in the amide I region. Furthermore, 2D-COS was additionally carried out using a modified mean normalization scheme, which has previously been used to apply 2D-COS to IR spectra of the amide I region to analyze protein unfolding.^32,33^ The resonance Raman spectra were vector normalized to account for different enhancement factors depending on the state of degradation. 2D correlation analysis for all spectra was carried out using in-house developed methods that have been described in detail elsewhere.^34,35^

## Results

### Raman Homo Two-Dimensional Correlation Spectroscopy

**Figure 2.**
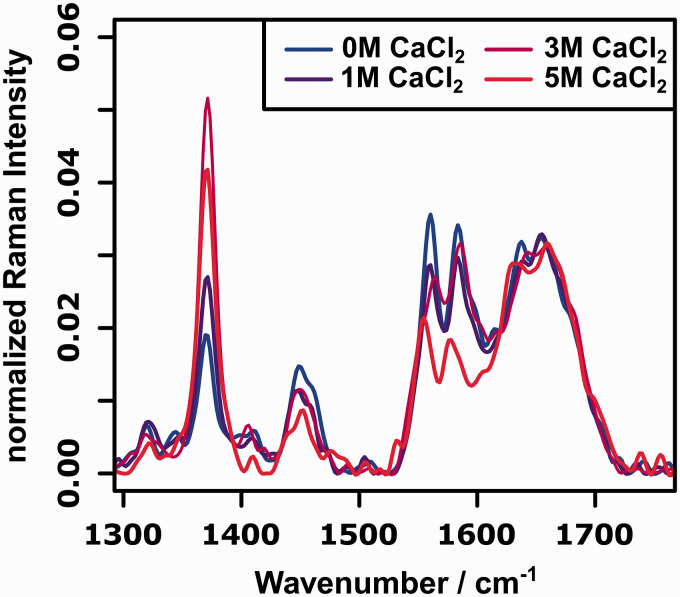
FT-Raman spectra ( $\lambda_{\text{exc}}$ = 1064 nm) of cytochrome c between 1000 and 1750 cm^−1^ for selected concentrations of CaCl_2_.

Figure 2 shows the FT-Raman spectra of cytochrome c for selected concentrations of CaCl_2_. The most prominent changes in the spectra can be observed around 1370 (amide-S band of protein backbone),^36^ 1450 (aromatic moieties), and 1550 cm^−1^ (heme-centered modes).^28^ The heme-centered modes as well as the aromatic moieties decrease in intensity upon addition of CaCl_2_. For the heme-centered modes, an additional slight shift in the wavenumber positions can be observed for very high concentrations of CaCl_2_. These changes are caused by structural changes of the heme moiety due to a change in secondary structure as will be discussed later for the resonance Raman spectra.

The changes in the amide-S band provide first insights into the secondary structure changes. It is well documented that the intensity of this band, which is assigned to a C α –H deformation, correlates inversely to the ratio of α -helices in the polymer.^36^ This is an expected behavior as upon denaturation the ratio of α -helices decreases. Interestingly, the intensity of the amide-S band decreases for very high concentrations of the denaturating agent. This observation, i.e., increase in intensity for increasing concentrations of denaturating agent followed by a decreasing intensity for very high concentrations of CaCl_2_ is due to a special property of cytochrome c, which undergoes a distinct meta-stable molten globule state during denaturation.^37,38^ The structural properties of this meta stable state differ from those of the natural as well as the fully denaturated form, which explains the decrease of intensity at 1370 cm^−1^ for very high ionic strengths.

For determining the secondary structure of the protein, the amide I region (1630–1700 cm^−1^) is the region of interest.^2^ Since the wavenumber position of the amide I vibration is sensitive to the protein conformation, this region can be used to directly probe the changes in secondary structure. However, as can be seen from the Raman spectra depicted in Fig. 2, the changes in this amide I wavenumber region are very subtle. Since the wavenumber positions of the amide I vibrations for the different secondary structures are very similar and the native protein consists of a mixture of α -helices and unordered turns (Fig. 1), the spectral signature does not change significantly during denaturation. In order to account for this, the amide I wavenumber region is usually deconvoluted by fitting Voigt profiles that correspond to the amide I vibrations expected for the pure secondary structural features.^2,9^ Here, we will show, that for the data sets recorded in this study, 2D-COS provides a model free alternative to analyze the amide I region.

**Figure 3.**
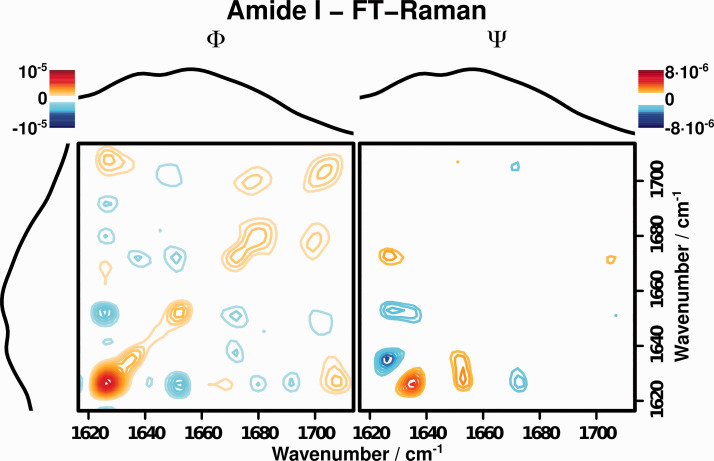
FT-Raman 2D-correlation spectra of the amide I region.

Figure 3 depicts the synchronous (Φ) and asynchronous (Ψ) correlation spectra of the amide I wavenumber region of cytochrome c for CaCl_2_ concentrations between 0 and 5 M after area normalization. Observed correlation patterns can be found in Table I. Compared to the one-dimensional (1D) spectra shown in Fig. 2, the band can be resolved in multiple components by applying 2D-COS to the data set (Table II). Four regions of strong changes can be identified: 1625 (aromatic vibration), 1655 (ordered α -helix), 1668 (random coil), 1678 (unordered helix), and 1702 cm^−1^ (random coil).^9^ The aromatic vibration does not directly depend on the secondary structure and can therefore be disregarded for further analysis.

**Table I.** Correlation patterns observed in the FT-Raman 2D-correlation spectra of the amide I region of cytochrome c.

Position (cm^−1^)	Sign syn.	Sign asyn.	Assignment
1655, 1668	−	0	α -Helix, rand. coil
1655, 1703	−	0	α -Helix, rand. coil
1668, 1678	+	0	Rand. coil, unord. helix
1678, 1703	+	0	Unord. helix, ran. coil
1625, 1655	+	−	Aromatic moiety, α -Helix

**Table II.** Relevant bands and assignment to functional moieties in the FT-Raman spectra of cytochrome c.

Band position (cm^−1^)	Assignment
1370	Amide-S^36^
1450	Aromatic vibrations
1550	Heme-centered modes^28^
1625	Aromatic vibrations
1655	Amide I, ordered α -helix^9^
1668	Amide I, random coil^9^
1678	Amide I, unordered helix^9^
1702	Amide I, random coil^9^

The 2D correlation analysis of the other vibrations allows for an easy characterization of the observed conformational changes of cytochrome c upon denaturation: α -helices, which are greatly stabilized by hydrogen bonds are transformed to a random coil structures or are at least disordered. This denaturation behavior corresponds to the negative signs at (1655, 1668) cm^−1^ and (1655, 1702) cm^−1^ in Φ, which according to Noda’s rules indicates changes in different directions.^15^ The absence of asynchronous correlation intensity for these patterns furthermore shows that these changes happen almost synchronously, which points to the fact that they stem from the same process. Significant asynchronous correlation intensity can only be found for the correlations with the aromatic vibration at 1625 cm^−1^. According to Noda’s rules, this band changes before the changes in secondary structure. This may be attributed to the fact that during the denaturation of cytochrome c, first a selection of amino acids near the porphyrin moiety is unfolded,^39^ thereby changing the aromatic vibrations in the covalently attached rings of the porphyrin. Since this process only targets a very small region of the protein, it does not change the secondary structure and therefore the amide I band significantly.

The synchronous 2D correlation spectrum obtained using the modified mean normalization scheme (Fig. S1, Supplemental Material) shows similar patterns for the amide I region. It is still possible to deconvolute the amide I into three distinct regions (1655 ( α -helix), 1678 (unordered helix), 1705 cm^−1^ (random coil)), which show the expected correlation pattern (negative cross-correlation between α -helix and the unordered states, positive cross-correlation between both unordered states). Using this normalization approach, the second random coil vibration at 1668 cm^−1^ cannot be clearly distinguished, although an asymmetry of the autopeak at 1678 cm^−1^ to lower wavenumbers is visible.

On the other hand, the asynchronous 2D correlation spectrum differs from the spectrum obtained by area normalization in that cross-peaks between the different amide I species are visible as well. These cross-peaks at (1655, 1680), (1655, 1703), and (1680, 1705) cm^−1^ have the same sign compared as the cross-peaks in the synchronous spectrum. This indicates that the transformation between the different protein conformations is not completely synchronous, but also follows a distinct pathway. The band at 1655 cm^−1^ assigned to α -helices first decreases in intensity, while the bands at 1678 cm^−1^ (unordered helix) and 1703 cm^−1^ (random coil) lag slightly behind. Furthermore, the changes to the unordered helix band also occur slightly before the changes to the band assigned to the random coil structure.

This asynchronicity matches the expected pathway for protein unfolding: The ordered helices first start to lose some of their order before being transformed to completely random, coil-like structures. Therefore, these correlation patterns give further insight into the pathway of unfolding observed in cytochrome c.

### Resonance Raman Homo Two-Dimensional Correlation Spectroscopy

**Figure 4.**
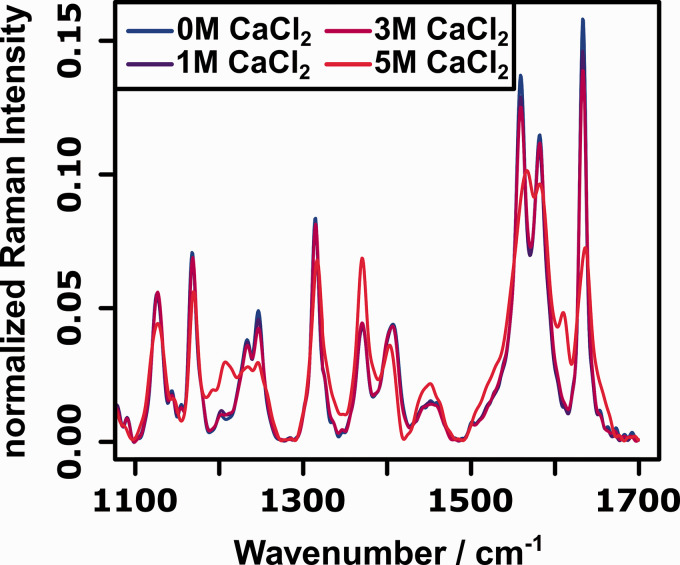
Resonance Raman spectra ( $\lambda_{\text{exc}}$ = 532 nm) of cytochrome c between 1000 and 1750 cm^−1^ for selected concentrations of CaCl_2_.

Figure 4 depicts a selection of the resonance Raman spectra of cytochrome c for different CaCl_2_ concentrations. Compared to the changes observed in the non-resonant FT-Raman spectra (Fig. 2), the changes in the resonance Raman spectra are much more pronounced, because the heme center undergoes larger structural changes as compared to the protein backbone upon denaturation. Furthermore, it can be seen that the observed spectral changes are very subtle up to a concentration of 3 M CaCl_2_, while the resonance Raman spectrum recorded for 5 M CaCl_2_ differs drastically from the remaining spectra. The strongest spectral changes upon addition of CaCl_2_ can be observed between 1200 and 1220 cm^−1^ ( δ C–H), 1360 and 1410 cm^−1^ (C α –S),^40^ and in the region between 1550 and 1650 cm^−1^. The latter wavenumber region contains several Raman bands, which are sensitive to the size, oxidation-, and spin state of the heme center and are therefore ideally suited to investigate the molecular changes within the active center (Table III).^29^

**Table III.** Relevant bands and assignment to functional moieties in the resonance Raman spectra of cytochrome c.

Band position (cm^−1^)	Assignment
1200–1220	δ C–H
1410	C α –S^40^
1560	C–C, oxidation state, core size^27^
1570	C–C, Fe high spin^28^
1582	C–C, Fe low spin^28^
1610	C–C, Fe high spin^28^
1530	C–C, Fe low spin^28^

**Figure 5.**
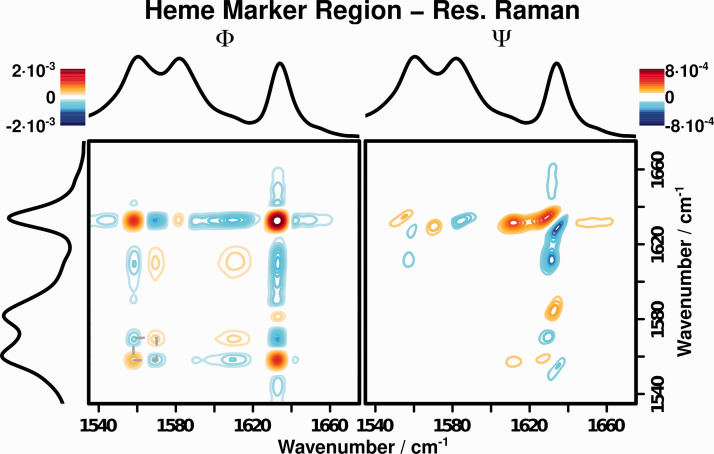
Resonance Raman ( $\lambda_{\text{exc}}$ = 532 nm) 2D-COS of the heme marker region.

Figure 5 depicts the synchronous and asynchronous 2D-COS spectra in this wavenumber region for the resonance Raman spectra recorded in the 2 to 5 M CaCl_2_ concentration range. The observed correlation patterns can be found in Table IV. A number of correlation patterns can be seen in Φ which correspond to literature-known band positions for heme-proteins in various states: the band at 1560 cm^−1^ is a combined oxidation state and core size marker,^27^ the bands at 1570, 1582, 1610, and 1632 cm^−1^ are all spin-state marker bands.^28^ The bands at 1582 and 1630 cm^−1^, which can be seen in the native state of the cytochrome c (Fig. 4), are indicative for a low-spin configuration of the heme center, while the resonance Raman bands at 1570 and 1610 cm^−1^ correspond to the same vibrations for a high-spin configuration.

**Table IV.** Correlation patterns observed in the resonance Raman 2D-COS of the heme marker region of cytochrome c.

Position (cm^−1^)	Sign syn.	Sign asyn.	Assignment
1560, 1570	−	0	Core size, Fe high spin
1560, 1610	−	0	Core size, Fe high spin
1560, 1630	+	− (low edge)	Core size, Fe low spin
1570, 1610	+	−	Fe high spin, Fe high spin
1570, 1630	−	+ (low edge)	Fe high spin, Fe low spin
1582, 1682	+	−	Fe low spin, Fe low spin
1610, 1630	−	+ (low edge)	Fe high spin, Fe low spin

The correlation patterns visible in Fig. 5 clearly show positive correlations between resonance Raman bands characteristic for each spin state and a negative correlation between bands characteristic for different spin states, showing the (partial) transition of the cytochrome c from low to high spin upon denaturation. Furthermore, a correlation with the size marker band at 1560 cm^−1^ is visible that decreases slightly in intensity and broadens to lower wavenumbers, as expected for larger core sizes.^27^ This broadening is not directly visible on the main diagonal of the 2D-COS plots, since it only slightly changes the intensity, but can indirectly be inferred from the correlation with the strong spin marker band at approximately (1545, 1630) cm^−1^ in Φ.

Ψ provides further insight into the changes of the heme center upon denaturation. Significant asynchronous correlation intensity is visible around the spin marker band at 1630 cm^−1^. This band slightly shifts to higher wavenumbers for higher CaCl_2_ concentrations. This observation is consistent with the known behavior of cytochrome c that a reconfiguration from the low- to the high-spin state (which is not complete, even at 5 M CaCl_2_) happens through an intermediary state that is still low-spin configured.^25,26^ This intermediary state differs from the natural state, hence its spin-marker band is slightly shifted. According to Noda’s rules, Ψ shows that this shift happens before the appearance of the high-spin marker band at 1610 cm^−1^, showing the intermediary nature of this state.

Furthermore, the correlation patterns at (1560, 1610) cm^−1^ show that the geometry change (indicated by the peak at 1560 cm^−1^) happens before the appearance of the high-spin state marker band at 1610 cm^−1^, which is also consistent with what one would expect: since the transition in spin state is caused by the displacement of the Met ligand by a water ligand,^26^ the geometry has to change, before the spin state changes.

### (Resonance) Raman Hetero Two-Dimensional Correlation Spectroscopy

**Figure 6.**
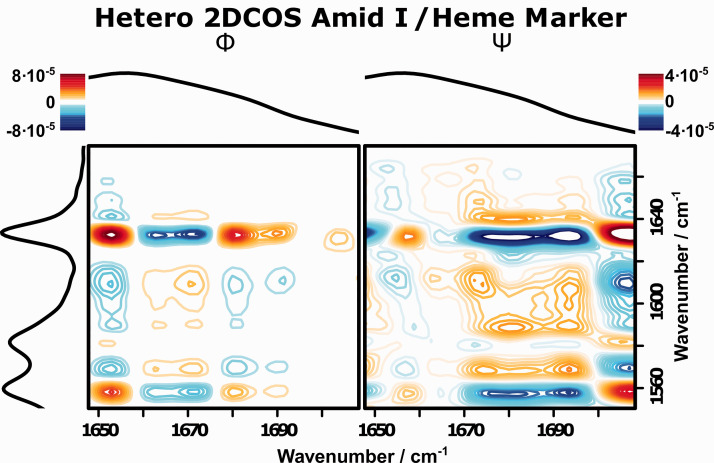
Hetero resonance Raman ( $\lambda_{\text{exc}}$ = 532 nm)/FT-Raman 2D-COS of the heme marker (resonance Raman, *y*-axis) and amide I (FT-Raman, *x*-axis) regions.

While the just presented separate 2D-COS analysis of the resonance and FT-Raman spectra already reveals much of the structural changes occurring upon cytochrome c denaturation, the ability to correlate the observed changes within both spectroscopic methods via a 2D hetero correlation approach is the most powerful way to apply 2D-COS. Such a hetero 2D-COS analysis allows an investigation of the relationship between the structural changes of the protein backbone, observed with FT-Raman, with the structural changes in the heme center, observed with resonance Raman. Figure 6 displays the hetero 2D-COS spectra of the wavenumber regions discussed in the sections above. Both Φ and Ψ show a number of correlation patterns that shed even more light in understanding the structural changes occurring in cytochrome c upon denaturation (Table V).

**Table V.** Correlation patterns observed in the hetero (resonance-) Raman 2D-COS of the heme marker and amide I regions of cytochrome c.

Position (cm^−1^)	Sign syn.	Sign asyn.	Assignment
1658, 1570	−	−	α -Helix, Fe high spin
1658, 1580	+	+	α -Helix, Fe low spin
1658, 1610	−	−	α -Helix, Fe high spin
1658, 1630	+	+	α -Helix, Fe low spin
1668, 1570	+	+	Rand. coil, Fe high spin
1668, 1580	−	−	Rand. coil, Fe low spin
1668, 1610	+	+	Rand. coil, Fe high spin
1668, 1630	−	−	Rand. coil, Fe low spin
1675, 1570	+	+	Unord. helix, Fe high spin
1675, 1580	−	−	Unord. helix, Fe low spin
1675, 1610	+	+	Unord. helix, Fe high spin
1675, 1630	−	−	Unord. helix, Fe low spin
1680, 1560	+	−	Unord. helix, core size
1690, 1560	+	−	Unord. helix, core size
1680, 1570	+	−	Unord. helix, Fe high spin
1690, 1570	+	−	Unord. helix, Fe high spin

The spin marker bands for the low-spin states at 1580 and 1630 cm^−1^ in the resonance Raman spectrum (*y*-axis) show positive correlation intensity with the secondary structure marker band for the ordered α -helices (1658 cm^−1^ in the FT-Raman spectrum, *x*-axis) and negative correlations with the marker bands for unordered states at 1668 and 1675 cm^−1^. The opposite holds true for the correlation patterns of the low-spin marker bands at 1570 and 1610 cm^−1^. Hetero 2D-COS is hence able to clearly show that the observed change in secondary structure, caused by the denaturation of the protein backbone, is directly correlated with a change of the heme spin state. For a system with unknown behavior, this would be a very strong hint to a causal relationship, which for the test system cytochrome c studied here to demonstrate the potential of hetero 2D-COS is well documented.

Ψ furthermore allows to probe the order of these changes. It can be seen that the sign for all the correlation patterns described above is the same in Ψ and Φ. Therefore, the changes on the *x*-axis (amide I region) happen before the changes on the *y*-axis (heme marker region). 2D-COS is thereby able to resolve the pathway through which the denaturation of cytochrome c commences. The secondary structure starts to change upon addition of even small quantities of CaCl_2_, but the changes are not strong enough to disturb the ligand sphere around the heme moiety in the active center of the protein. Only for higher concentrations are the structural changes of the protein structure strong enough that the Met ligand of the Fe-center gets displaced by a water ligand, which in turn triggers the transition from low- to high-spin states.

Additionally, the hetero 2D-COS reveals that further changes in the amide I region happen after the spin state has changed. The wavenumber region between 1680 and 1700 cm^−1^ on the *x*-axis shows correlation patterns in Φ that are opposite to those between 1665 and 1675 cm^−1^, while the sign in Ψ stays the same. According to Noda’s rules, a process that happens after the change in spin state must therefore be responsible for the changes in this spectral region. As discussed earlier, the transition of cytochrome c to its denaturated form happens via meta-stable states. This additional process can therefore be assigned to a change from these meta-stable states to a further denaturated form.

In the hetero 2D-COS case, the application of the modified mean normalization did not add any additional insights (Fig. S2, Supplemental Material). The only difference between the two normalization schemes is that the modified mean normalization shows only very weak correlation patterns for the random-coil amide I vibration at 1670 cm^−1^, as already observed in the homo FT-Raman 2D-COS—all other patterns and therefore conclusions are qualitatively the same for both normalization schemes.

The complete mechanism can therefore be described by the following sequence of events: Upon addition of CaCl_2_, the α -helices begin to transition to unordered helices and random coil-like structures. In this transition state, some of the heme centers are already in a high-spin configuration, but the equilibrium still lies on the side of the low-spin configuration. From this transition state, further addition of CaCl_2_ leads to further unfolding that change the band shape of the amide I region further and push the equilibrium of the spin states further to the side of the high-spin configuration. This mechanism cannot be easily identified from either analyzing the 1D spectra (Figures 2 and 4) nor the homo 2D-COS spectra (Figures 3 and 5), which clearly demonstrates the power of the hetero 2D-COS approach for the analysis of photoactive proteins.

## Conclusion

Here, we report on a Raman/resonance Raman hetero 2D-COS approach to investigate the denaturation behavior of cytochrome c. For this, we recorded FT-Raman ( $\lambda_{\text{exc}}$ = 1064 nm) and resonance Raman ( $\lambda_{\text{exc}}$ = 532 nm) spectra of cytochrome c in aqueous solutions of CaCl_2_ for a concentration range between 0 and 5 M. The addition of CaCl_2_ to cytochrome c leads to a defined denaturation of the protein and with it also a change of the functionality of the active heme moiety.

We could show that by using FT-Raman 2D-COS it is possible to resolve the highly overlapped amide-I region of the Raman spectrum without applying the traditional band fitting techniques. In comparison to these band fitting techniques, 2D-COS is a model free technique that does not require the specification of a number of bands or approximate wavenumber positions for these bands. The results show that the denaturation of cytochrome c from a highly ordered structure containing a large proportion of α -helices to an unorderded structure mainly consisting of random coil and unordered helices. Furthermore, by investigating the amide-S band, it was possible to identify the molten-globule transition state typical for cytochrome c denaturations.

By analyzing the resonance Raman spectra, we were able to fully resolve the changes in the active heme center of cytochrome c upon denaturation: the spin and core-size marker region between 1540 and 1650 cm^−1^ allowed us to follow the transition of the Fe-center from a low- to a high-spin configuration due to the displacement of stabilizing amino acid ligands. By employing 2D-COS, we were furthermore able to resolve the order of changes in the structure of the active center and could demonstrate that the spin state only changes after the porphyrin geometry has changed. Additionally, the presence of a high-/low-spin equilibrium in the transition from a low- to a high-spin configuration could be revealed that is not detectable from analyzing the 1D spectra alone.

The combination of both spectroscopic methods to a Raman/resonance Raman hetero 2D-COS spectrum then allowed us to directly elucidate the relationship between secondary structure and structure of the heme-moiety of cytochrome c: The discussed changes in secondary structure are directly correlated with the discussed changes in the active center, as expected for structural elements coupled through such a relationship. The order resolving properties of hetero 2D-COS made it furthermore possible to reveal the complete sequence of changes occurring in cytochrome c upon denaturation. Combining these spectroscopic results with studies into the functionality of a protein (i.e., catalytic activity, oxygen binding capacity) therefore offers a promising approach to elucidate structure–function relationships in proteins, which are not as well characterized as cytochrome c.

In conclusion, we could demonstrate that the combination of Raman and resonance Raman spectroscopy with 2D-COS offers a powerful tool to correlate secondary structure and active moiety in proteins with photoactive centers. This approach can in principle be transferred to an arbitrary combination of techniques with different specificities to different moieties, such as near- and mid-infrared spectroscopy. Paired with the non-invasive nature of these spectroscopic techniques and the minimal need for sample preparation, hetero 2D-COS is an ideal candidate to elucidate structure--function relationships of proteins in situ.

## Supplemental Material

sj-pdf-1-asp-10.1177_00037028211028916 - Supplemental material for Probing Protein Secondary Structure Influence on Active Centers with Hetero Two-Dimensional Correlation (Resonance) Raman Spectroscopy: A Demonstration on Cytochrome C Click here for additional data file. Supplemental material, sj-pdf-1-asp-10.1177_00037028211028916 for Probing Protein Secondary Structure Influence on Active Centers with Hetero Two-Dimensional Correlation (Resonance) Raman Spectroscopy: A Demonstration on Cytochrome C by Julian Hniopek, Thomas Bocklitz, Michael Schmitt and Jürgen Popp in Applied Spectroscopy

sj-pdf-2-asp-10.1177_00037028211028916 - Supplemental material for Probing Protein Secondary Structure Influence on Active Centers with Hetero Two-Dimensional Correlation (Resonance) Raman Spectroscopy: A Demonstration on Cytochrome C Click here for additional data file. Supplemental material, sj-pdf-2-asp-10.1177_00037028211028916 for Probing Protein Secondary Structure Influence on Active Centers with Hetero Two-Dimensional Correlation (Resonance) Raman Spectroscopy: A Demonstration on Cytochrome C by Julian Hniopek, Thomas Bocklitz, Michael Schmitt and Jürgen Popp in Applied Spectroscopy
