# Supplementary material for: Probing Protein Secondary Structure Influence on Active Centers with Hetero Two-Dimensional Correlation (Resonance) Raman Spectroscopy: A Demonstration on Cytochrome C
Source: Appl Spectrosc. 2021 Jul 9;75(8):1043–52. doi: 10.1177/00037028211028916 (PMC8320570; doi:10.1177/00037028211028916)
Supplement: sj-pdf-1-asp-10.1177_00037028211028916 - Supplemental material for Probing Protein Secondary Structure Influence on Active Centers with Hetero Two-Dimensional Correlation (Resonance) Raman Spectroscopy: A Demonstration on Cytochrome C [file sj-pdf-1-asp-10.1177_00037028211028916.pdf]

Probing Protein Secondary Structure Influence on Active Centers with Hetero 2D Correlation (Resonance-) Raman-Spectroscopy: A Demonstration on Cytochrome C - Supporting Information

Julian Hniopek[1,2], Thomas Bocklitz[1,3], Michael Schmitt[2], and Jürgen Popp[1,2,*]

[1]Department of Spectroscopy/Imaging, Leibniz-Institute of Photonic Technologies, Albert-Einstein-Straße 9, 07745 Jena, Germany

[2]Institute of Physical Chemistry & Abbe Center of Photonics, Friedrich Schiller University Jena, Helmholtzweg 4, 07743 Jena, Germany

[3]Department of Photonic Data Science, Leibniz-Institute of Photonic Technologies, Albert-Einstein-Straße 9, 07745 Jena, Germany

[*]juergen.popp@leibniz-ipht.de

Additional 2D Correlation Spectra using modified mean normalization

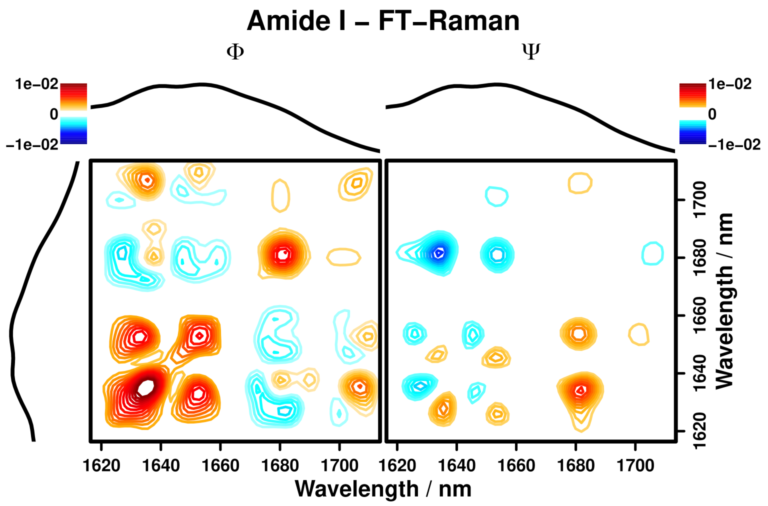

Figure 1: FT-Raman 2D-Correlation spectra of the amide I region using the modified mean normalization approach.

Table 1: Relevant correlation patterns observed in the FT Raman 2D correlation spectra of the amide I region of cytochrome c using the modified mean normalization approach.

Position / cm^{-1}	Sign Syn.	Sign Asyn.	Assignment
1655, 1680	-	-	α-helix, unord. helix
1655, 1703	-	-	α-helix, rand. coil
1680, 1703	+	+	unord. helix, rand. coil

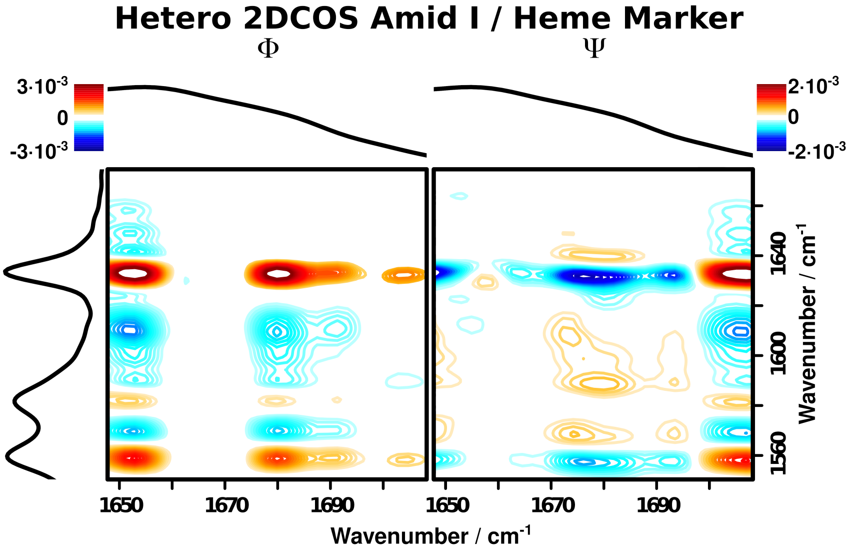

Figure 2: Hetero Resonance-Raman ($\lambda_{exc} = 532$ nm) / FT Raman 2D correlation spectra (using the modified mean normalization scheme) of the heme marker (resonance Raman, y-axis) and amide I (FT-Raman, x-axis) regions.

Table 2: Relevant correlation patterns observed in the hetero (resonance-) Raman 2D correlation spectra of the heme marker and amide I regions of cytochrome c using the modified mean normalization for the non resonant Raman spectra.

Position / cm^{-1}	Sign Syn.	Sign Asyn.	Assignment
1658, 1570	-	-	*alpha*-helix, Fe high spin
1658, 1580	+	+	*alpha*-helix, Fe low spin
1658, 1610	-	-	*alpha*-helix, Fe high spin
1658, 1630	+	+	*alpha*-helix, Fe low spin
1675, 1570	+	+	unord. helix, Fe high spin
1675, 1580	-	-	unord. helix, Fe low spin
1675, 1610	+	+	unord. helix, Fe high spin
1675, 1630	-	-	unord. helix, Fe low spin
1680, 1560	+	-	unord. helix, Core size
1690, 1560	+	-	unord. helix, Core size
1680, 1570	+	-	unord. helix, Fe high spin
1690, 1570	+	-	unord. helix, Fe high spin
